# Pulmonary Hemorrhage following Edge-to-Edge Mitral Valve Repair

**DOI:** 10.1155/2017/4854736

**Published:** 2017-06-19

**Authors:** Mirna B. Ayache, Myttle A. Mayuga, Chantal ElAmm, Guilherme Attizzani, Jordan Kazakov

**Affiliations:** ^1^Department of Medicine, Division of Pulmonary, Critical Care and Sleep Medicine, University Hospitals Cleveland Medical Center, Cleveland, OH, USA; ^2^Department of Medicine, Division of Cardiovascular Medicine, University Hospitals Cleveland Medical Center, Cleveland, OH, USA

## Abstract

Mitral valve repair with the MitraClip device has emerged as an effective treatment option for patients with severe mitral regurgitation and contraindications for surgical interventions. While the procedure is not known to cause pulmonary complications, we describe two cases of pulmonary hemorrhage following percutaneous mitral valve repair. The patients did well with supportive care and reinitiation of anticlotting agents was well tolerated after resolution of bleeding.

## 1. Introduction

Percutaneous edge-to-edge mitral valve repair (MVR) with the MitraClip device has emerged as an effective treatment option for patients with severe mitral regurgitation (MR) who are at prohibitive risk for surgery. The procedure is performed under general anesthesia with transesophageal echo guidance. Procedural success, described as a reduction in MR severity to grade 2+ or less, results in improvements in cardiac output and New York Heart Association (NYHA) class [1]. These findings are accompanied by a reduction in left ventricle preload, represented by a significant reduction in the pulmonary capillary wedge pressure and pulmonary arterial pressure [1, 2]. Most patients undergoing percutaneous MVR are extubated immediately after the procedure and have no significant pulmonary sequelae. Here we describe two cases of percutaneous MVR immediately followed by pulmonary bleeding in the left lung confirmed by bronchoscopy.

## 2. Case Presentation

**Figure 1:**
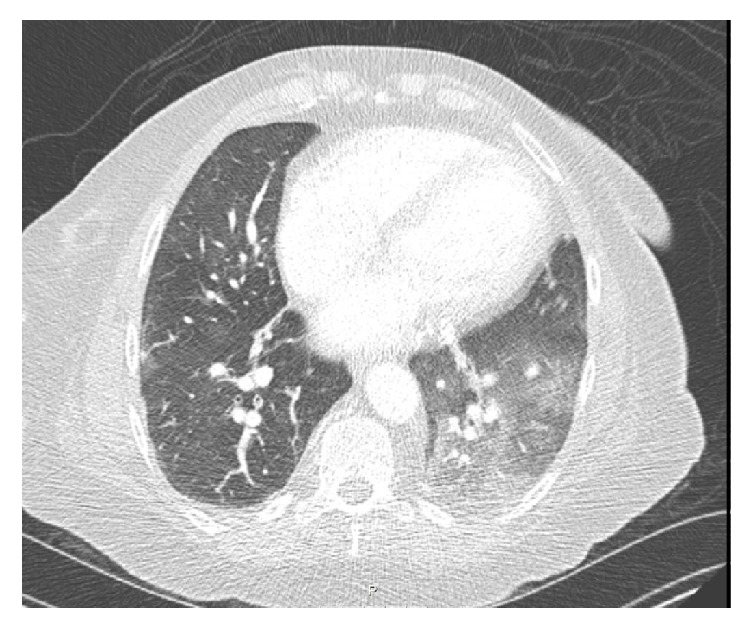
CT chest showing ground glass opacities predominantly on the left side (case 1).

The first case is a 55-year-old female with history of asthma, hypertension, end stage renal disease secondary to systemic lupus erythematosus (SLE) on hemodialysis, heart failure (HF) with preserved ejection fraction (EF), and severe MR who underwent successful elective mitral valve (MV) clipping with reduction in MR from 4+ to 2+. She developed bloody frothy secretions from the endotracheal tube after procedure. Hemoglobin after procedure was 3 g/dl lower than baseline which was attributed to right thigh hematoma at the vascular access. She was extubated and continued to have mild hemoptysis. CT chest was negative for pulmonary embolism (PE) and showed ground glass opacities (GGO) predominantly in the left lung (Figure 1). Bronchoscopy revealed bleeding coming from the left lung with positive bronchoalveolar lavage (BAL) for pulmonary alveolar hemorrhage (PAH) in the lingula. Hemoptysis resolved upon discontinuation of aspirin and clopidogrel. Dual antiplatelet therapy was restarted few days after hemoptysis resolution without evidence of pulmonary hemorrhage recurrence.

**Figure 2:**
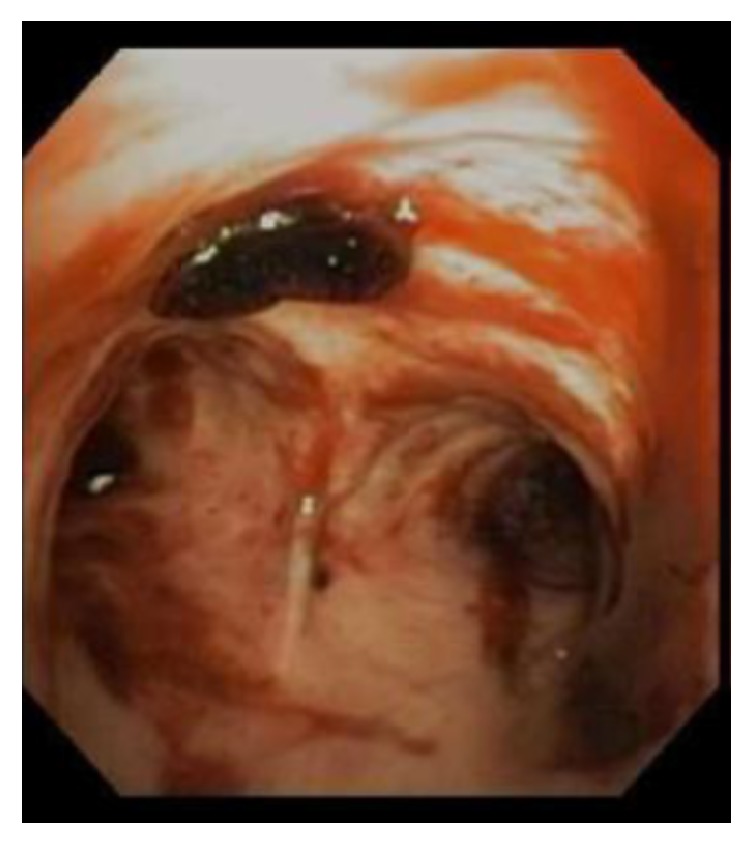
Bronchoscopy showing blood in the airways left more than right (case 2).

**Figure 3:**
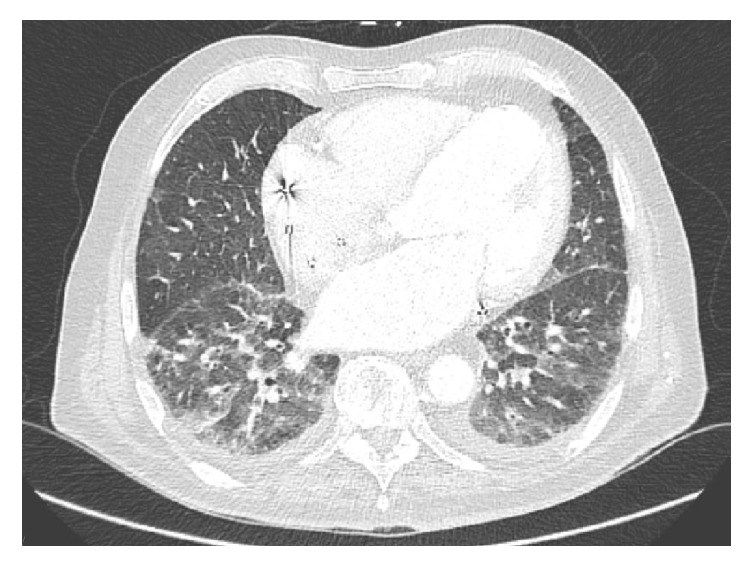
CT chest showing bilateral ground glass opacities consistent with congestive heart failure (case 2).

The second case is an 83-year-old male with a history of coronary artery disease, stage 3 chronic kidney disease, atrial fibrillations s/p ablation, HF with reduced EF, and severe MR who underwent successful elective MV clipping procedure with trace residual MR. The procedure was followed by hypotension requiring epinephrine drip and a large amount of blood was noted coming from the endotracheal tube. Bronchoscopy revealed blood in the airways left more than right (Figure 2), a large blood clot in the left main bronchus, a bleeding source likely left lower lobe (LLL), and negative BAL for PAH in the right middle lobe (RML). Hemoglobin dropped by 2 g/dl. CT chest angiography was negative for PE and showed bilateral lower lobes GGO present on previous imaging consistent with HF (Figure 3). Pressors were weaned off with volume resuscitation and the patient was successfully extubated with no further pulmonary bleeding. He was initially maintained on low dose aspirin. Clopidogrel and warfarin were added few days later and clopidogrel was discontinued when anticoagulation was therapeutic.

## 3. Discussion

To our knowledge, these are the first cases to describe pulmonary hemorrhage immediately following percutaneous MVR. In both cases, upper and larger lower airway sources of bleeding were excluded by visual inspection during bronchoscopy and PE was excluded by CT chest. In the first case, PAH was confirmed by increasing blood on three sequential lavage aliquots. Differential diagnosis includes PAH with capillaritis such as Goodpasture syndrome, antineutrophil cytoplasmic antibody positive vasculitis, and connective tissue disorders [3]. However, there was no clinical or laboratory suspicion for acute inflammatory process. Moreover, the patient's hemoptysis resolved without targeted treatment for capillaritis. Bland pulmonary hemorrhage can be caused by antiplatelet therapy [4] and MR [5–7]. However, it is not clear why it would manifest right after the procedure predominately in the left lung and before clopidogrel loading. The second case represents a more dramatic pulmonary hemorrhage immediately following the procedure. Bronchoscopy showed a large blood clot in the left main bronchus with no evidence of airway bleeding after clot removal. BAL was negative for PAH in the RML and there was no clinical suspicion of systemic disease causing capillaritis. Like the first case, MR/CHF and aspirin therapy can result in pulmonary hemorrhage but the onset right after the procedure and severity of unilateral lung bleeding suggest that the procedure was a causative or precipitating factor. However, pulmonary hemorrhage after percutaneous mitral valve repair has not been noted by highly experienced interventional cardiology expertise and therefore further case descriptions of any similar adverse events will be helpful in solving this dilemma. Although hypothetical with no objective confirmatory evidence, potential theories are worth mentioning that would explain pulmonary hemorrhage after percutaneous mitral valve repair.

During the MitraClip procedure, a stiff wire is used to cross the interatrial septum, providing a rail over which the steerable guide catheter is advanced into the left atrium. It is common practice to create a “J” tip on soft end of this stiff wire, creating an atraumatic tip that is then advanced into a left pulmonary vein for anchoring. While there are no prior studies describing pulmonary vein complications during a MitraClip procedure (such as pulmonary vein dissection), hemoptysis has been described immediately after dilation of the left upper pulmonary vein during percutaneous pulmonary vein stenting [8]. The occurrence of immediate pulmonary hemorrhage primarily in the left lung supports a theory of acute, transient left pulmonary vein obstruction. More studies are needed to assess this more systematically.

Another contributing factor may be the effect of eccentric MR jets on the pulmonary veins. Despite overall procedural success after MitraClip, an eccentric residual MR jet may remain. Quantification of the severity of MR with eccentric or multiple jets can be challenging, and the significance of these residual jets may be underappreciated. While one prior study failed to demonstrate an association between jet direction and pulmonary vein patterns [9], it is conceivable that an unrecognized, severe eccentric jet could flood one or more pulmonary veins even transiently, resulting in unilateral or unilobar pulmonary hypertension, edema, and hemorrhage. Further studies are warranted to examine whether this phenomenon exists, and whether it could have a significant impact on pulmonary hemodynamics.

In conclusion, we describe two cases of pulmonary hemorrhage in the left lung immediately following percutaneous mitral valve repair. The patients did well with supportive care and reinstitution of anticlotting agents was well tolerated.
